# Variation in winter site fidelity within and among individuals influences movement behavior in a partially migratory ungulate

**DOI:** 10.1371/journal.pone.0258128

**Published:** 2021-09-30

**Authors:** Timothy J. Fullman, Brian T. Person, Alexander K. Prichard, Lincoln S. Parrett

**Affiliations:** 1 The Wilderness Society, Anchorage, Alaska, United States of America; 2 Department of Wildlife Management, North Slope Borough, Utqiaġvik, Alaska, United States of America; 3 ABR, Inc.–Environmental Research & Services, Fairbanks, Alaska, United States of America; 4 Alaska Department of Fish and Game, Fairbanks, Alaska, United States of America; Universita degli Studi di Sassari, ITALY

## Abstract

Many animals migrate to take advantage of temporal and spatial variability in resources. These benefits are offset with costs like increased energetic expenditure and travel through unfamiliar areas. Differences in the cost-benefit ratio for individuals may lead to partial migration with one portion of a population migrating while another does not. We investigated migration dynamics and winter site fidelity for a long-distance partial migrant, barren ground caribou (*Rangifer tarandus granti*) of the Teshekpuk Caribou Herd in northern Alaska. We used GPS telemetry for 76 female caribou over 164 annual movement trajectories to identify timing and location of migration and winter use, proportion of migrants, and fidelity to different herd wintering areas. We found within-individual variation in movement behavior and wintering area use by the Teshekpuk Caribou Herd, adding caribou to the growing list of ungulates that can exhibit migratory plasticity. Using a first passage time–net squared displacement approach, we classified 78.7% of annual movement paths as migration, 11.6% as residency, and 9.8% as another strategy. Timing and distance of migration varied by season and wintering area. Duration of migration was longer for fall migration than for spring, which may relate to the latter featuring more directed movement. Caribou utilized four wintering areas, with multiple areas used each year. This variation occurred not just among different individuals, but state sequence analyses indicated low fidelity of individuals to wintering areas among years. Variability in movement behavior can have fitness consequences. As caribou face the pressures of a rapidly warming Arctic and ongoing human development and activities, further research is needed to investigate what factors influence this diversity of behaviors in Alaska and across the circumpolar Arctic.

## Introduction

Migration is a widely exhibited behavior among diverse taxa, including invertebrates, birds, mammals, reptiles, and fish [1–4]. Potential benefits of migration include escape from seasonally harsh environmental conditions, access to resources that vary over space and time, and reduced pressure from predators, disease, or parasites [5–8]. To obtain these benefits, migrants must endure the energetic costs of long-distance movement [9], bear the risk of navigating through unfamiliar landscapes [10], and have morphological capabilities sufficient to support migratory movements [7]. For some species, exposure to predation risk, hunting pressure, pathogens, and anthropogenic or natural barriers may be greater for migrants than resident individuals [8,11,12]. This balance of costs and benefits can influence fitness rates of animals [13,14], leading to tradeoffs in migratory versus residency behavior.

It is increasingly apparent that fitness tradeoffs resulting from different migratory behaviors lead to behavioral variability not just among species and populations, but within them. Partial migration–in which some individuals in a population migrate while others do not–is common among many migratory taxa [15,16], though not ubiquitous [17]. Even within partially migratory populations, simple classification into migrant and resident behavior is complicated by a wide array of migration behaviors, such as variability in the distance of migration [11] and decisions about when, where, and whether to migrate [18]. These can lead to fitness differences among individuals that adopt different strategies [11,13]. Fluctuating environmental conditions may further influence behavioral choices and their relative fitness impacts [13]. For example, conditional migration has been reported in some ungulate species, in which environmental conditions such as winter severity in a given year influence the likelihood that individuals migrate [19]. Such factors can result in changes in the prevalence of different behaviors within partially migratory populations [13].

Though iconic and important for many ecological processes [2,20,21], long-distance migrations are becoming imperiled across the globe [7,22–24]. Disruption of migration often has been linked to significant declines in populations [7]. Large mammalian herbivores feature many of the best-known and longest-distance terrestrial migrations in the world [25]. Potential threats to migration are especially concerning for this group of species, as recent work has highlighted the heightened extinction risk of large herbivores [26]. Better understanding the set of traits that allow for migration, such as navigational ability, timing of movements, site fidelity, sociality, and structural adaptations–the so-called “migration syndrome” [7,27,28]–as well as the degree of flexibility in those traits at individual and population levels, provides opportunities to comprehend natural variation in migratory species. Variability in migratory movements increases challenges for conserving species, as it tends to expand the scope of conservation efforts and the need to coordinate across management entities, as well as creating situations where the effectiveness of actions taken in one area may depend on those taken in other areas [29].

We investigate migratory flexibility in one of the world’s longest terrestrial migrants, barren ground caribou (*Rangifer tarandus granti*) [25], focusing on the partially migratory Teshekpuk Caribou Herd (TCH) in northern Alaska [30]. We focus primarily on migration and wintering areas, complementing prior research describing calving distribution and summer resource use for this herd [30–32]. Choice of wintering area by individuals of the TCH influences selection of migration routes and has implications for interactions of caribou with human activity including oil and gas exploration, development, hunting, and other activities by residents of local communities. Anthropogenic activity within portions of the herd range increases during winter, including snow machine travel and vehicle traffic along compacted snow roads used for community transportation and along ice roads used for energy exploration and construction.

Our primary objectives were to: 1) classify movement behavior of individual caribou to determine the degree of partial migration (percentage migrants, residents, and other behavior) over time, 2) characterize migration dynamics, identifying patterns of timing, distance, destination, and directedness of migration, and 3) analyze the degree of fidelity to wintering areas accessed by individuals within the herd. We build upon research documenting TCH seasonal distributions and movement timing prior to oil and gas development in the herd range [30] to provide a baseline to compare future migration patterns under natural environmental variation, infrastructure development, and climate change.

## Methods

### Study area and species

**Fig 1:**
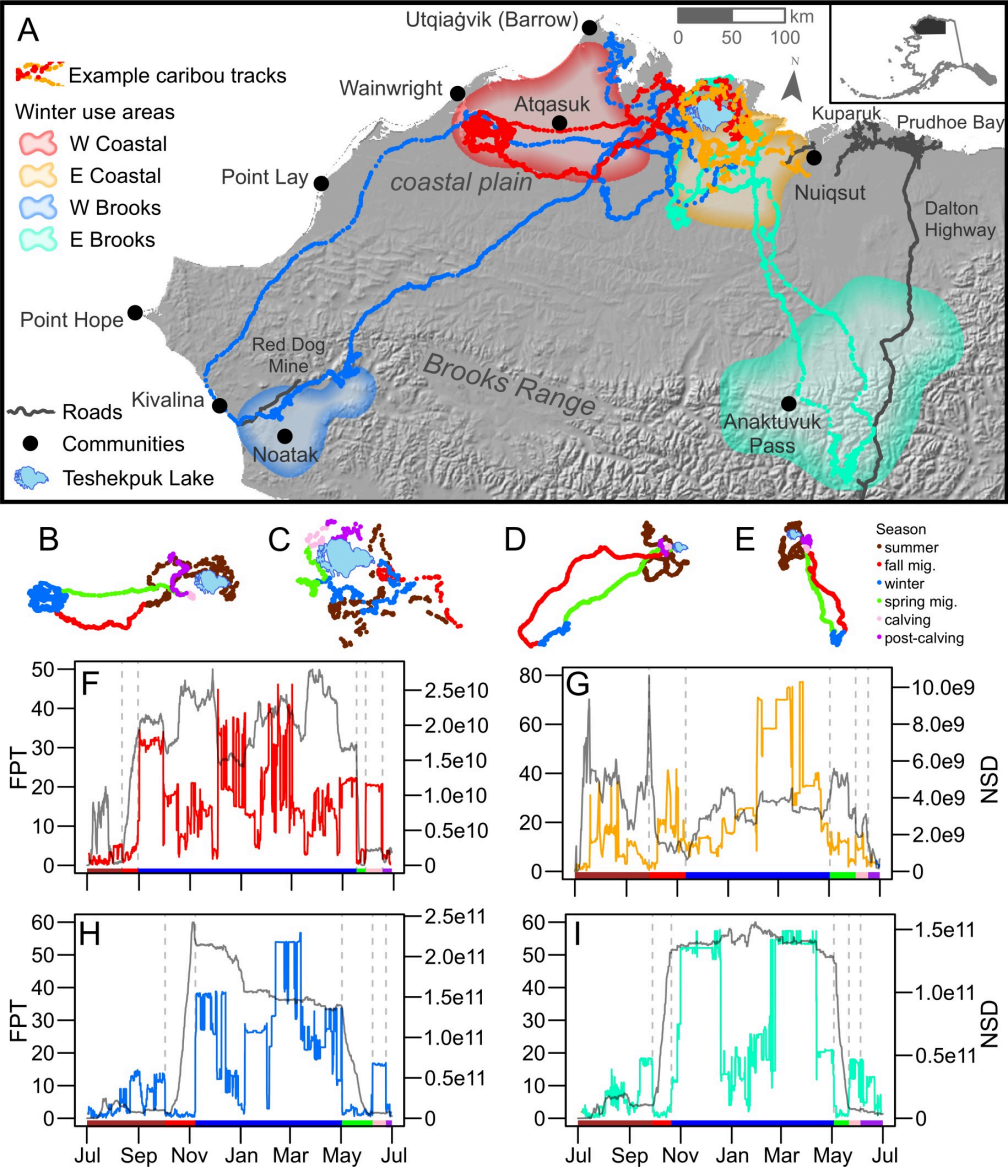
Example caribou movement data for four individuals from the Teshekpuk Caribou Herd (TCH) in northwestern Alaska. Caribou of the TCH display a variety of movement patterns, including use of four wintering areas. (A) One representative caribou-year of data is depicted for each wintering area. (B-E) Individual paths for the four individuals in panel A are shown divided by season. Teshekpuk Lake, the vicinity of most calving for the TCH, is shown in each panel for reference, with size varying relative to movement distance. (F-I) Corresponding first passage time–net squared displacement (FPT-NSD) plots for caribou overwintering in the (B,F) western coastal plain (W Coastal), (C,G) eastern coastal plain (E Coastal), (D,H) western Brooks Range (W Brooks), and (E,I) eastern Brooks Range (E Brooks). Seasonal periods in panels B-E are replicated along the x-axis of each corresponding FPT-NSD plot, with vertical dashed grey lines indicating season breaks. Panels F-I depict the FPT in the color matching the corresponding movement data in panel A and NSD in grey.

The TCH is one of four caribou herds that calve on the North Slope of Alaska, along with the Western Arctic Herd, Central Arctic Herd, and Porcupine Caribou Herd. The TCH numbers approximately 56,000 caribou [33]. The herd primarily calves around Teshekpuk Lake in northwestern Alaska (Fig 1) [30–32], though some calving has been noted farther west [34]. After calving, the herd clusters along the coast and in riparian areas seeking relief from mosquitoes (*Culex* spp.) and oestrid flies (*Hypoderma* spp. and *Cephenemyia* spp.) before spreading out to forage across the arctic coastal plain [30,31]. Unlike the other three large migratory herds in northern Alaska, the majority of the TCH remains on the coastal plain year-round [30,35]. Some TCH caribou remain resident near Teshekpuk Lake, others make a relatively short-distance migration to wintering areas on the western coastal plain, while part of the herd migrates from the coastal plain to overwinter in the Brooks Range mountains and areas farther south [30].

Much of the TCH range is undeveloped. While there are small communities across the herd range, none are connected by permanent roads and most lie near the coast (Fig 1). The oil and gas industrial complexes of Prudhoe Bay and Kuparuk primarily lie to the east of the herd range. Oil developments have moved into the eastern portion of the herd range in recent years, and additional development extending west from existing infrastructure has been permitted [36]. Other development in the herd range includes the Dalton Highway in the east that connects Prudhoe Bay to Fairbanks and communities further south, and the Delong Mountain Transportation System, servicing the Red Dog Mine in the west (Fig 1).

### Data collection and preparation

All caribou were captured using a manually fired net gun from an R44 helicopter before being restrained with blindfolds and hobbles for measurement and collaring. Caribou aged 13 months and older were captured in late June and early July, often near Teshekpuk Lake. Captured caribou were fitted with a collar containing a conventional very high frequency (VHF) radio-transmitter and a GPS-linked transmitter (various TGW- models; Telonics, Mesa, AZ). Collars were adjusted to allow for growth and to minimize rubbing. Recaptures occurred at 1-5-year intervals based on expected battery life of the collar. All captures were conducted under Alaska Department of Fish and Game Institutional Animal Care and Use Approval #2007–13 and subsequent renewals.

We received location and mortality data through polar-orbiting satellites transmitted through command and acquisition stations to ARGOS data processing centers [37]. Location data spanned 2004–2016, however we excluded periods in which individuals had their GPS collars replaced with a Platform Terminal Transmitter (PTT) collar due to lower fix rates and positional accuracy. For example, all caribou had their GPS collars replaced with PTT collars in 2005, resulting in a lack of GPS records for the July 2005 –June 2006 period. Data filtering removed locations that were duplicated, post-mortality, or presumed erroneous based on the combination of distance, rate, and angle [38]. We then divided data for each caribou into analysis-years stretching from July 1 of one year to June 30 of the subsequent year. This encapsulates a time from after calving, when caribou typically gather while seeking relief from insects, through a full year until shortly after the next calving season, which coincides with annual collaring efforts. We refer to one caribou-year of data as a single analysis-year of data for one individual caribou.

To retain only relatively complete caribou-years, we filtered the data by excluding individual caribou-years with a duration less than 290 days [39]. Furthermore, we removed caribou-years with gaps in location information greater than 2 consecutive weeks to ensure there were sufficient records for each month. However, individuals whose records terminated early, either due to mortality or collar failure, were retained as long as their records met the 290-day threshold. We removed five caribou-years in which a caribou calved with a different herd and then followed that herd’s predominant movement patterns, or spent most of the year with that herd, as these did not reflect movement patterns for the TCH [30]. In Alaska, caribou herds are identified based on fidelity to calving grounds for ecological and management purposes [40,41], although caribou do sometimes switch between herds both temporarily and for extended periods [35]. We retained one caribou-year in which the individual spent the calving period near the Western Arctic Herd calving area but then rejoined the main TCH area [35].

Telemetry collars recorded caribou locations at varying pre-programed fix intervals ranging from locations every 2 hours to locations every 12 hours. Some collars featured variable fix rates throughout the year with certain periods programed at a coarser fix interval (e.g., during winter) and others at a finer fix interval (e.g., around calving). Each collar’s records were standardized to its coarsest time interval across the entire analysis-year.

### Classifying movement behavior

We classified caribou movement behavior into a set of possible strategies (i.e., residency, migration, or other movement) and used characteristics of the movement trajectories to define seasonal periods. There are multiple ways to characterize animal movement behavior, leading some researchers to recommend comparing results using multiple classification methods [39]. We evaluated four potential methods for classifying movement behavior of caribou: home range overlap [39], latent state modeling of net squared displacement [42], mechanistic range shift analysis [18], and first-passage time–net squared displacement [43,44]. After comparing results of the four approaches, we found only the first passage time–net squared displacement approach (FPT-NSD) suitable for our purposes of characterizing caribou movement and identifying seasonal use (see S1 Appendix). Only FPT-NSD could accommodate the variety of movement types displayed within the TCH, such as movement bursts interspersed with largely sedentary periods, occasional movement bouts during the winter, and the vast size of the winter range.

The FPT-NSD approach combined movement-based and location-based data to identify seasonal movement patterns [43,44]. First passage time (FPT) identified periods of tortuous movement and those characterized by faster, more linear movement [45]. We subdivided the movement paths for each caribou-year into groups with similar FPT values using a segmentation process [46] to indicate breakpoints in movement behavior. The segmentation process often indicated more breakpoints than just those surrounding migration (e.g., the summer period, prior to fall migration, might be subdivided into three segments: high movement to reach insect relief habitat, clustering during insect relief, and high movement post-insect relief but before migration). We manually reviewed candidate breakpoints for each individual to classify seasonal movement periods using a combination of the individual’s FPT values, net squared displacement (NSD) values that signal changes in movement areas [47], and visual analysis of segmented locations. For additional details regarding application of the FPT-NSD approach, please see S1 Appendix.

We conducted all analyses using the statistical software R (version 3.4.0) [48]. We calculated FPT and performed FPT segmentation using the adehabitatLT package [49] and manually calculated two sets of NSD values, one based on the first recorded location of each analysis-year and the other on first day of winter behavior. From the segmented data, we assigned movement classifications (migrant, resident, and other). To count as migration a caribou had to exhibit discrete summer and winter ranges and a general there-and-back-again return movement, but we did not employ distance thresholds, as this varied widely among individuals. Resident behavior did not exhibit discrete summer and winter ranges and the caribou remained in the general vicinity of Teshekpuk Lake.

### Migration characterization

For caribou classified as migrants by the FPT-NSD approach, we recorded the start and end dates of fall and spring migration, as well as the duration and distance of migration. We recorded both Euclidean distance between the start and end points of migration and path distance, summing the distances between consecutive locations during the migratory period. The former approach is common in analyses of migration (e.g., [25,44]), while the latter approach estimates the cumulate distance traveled [25] and may be especially relevant when migration includes large looping movements, as was often seen for the TCH. Path distance is influenced by fix interval between locations [38,50], so we calculated both distances on a standardized dataset in which all caribou-years contributed at most two observations per day, corresponding to the coarsest fix interval in our dataset (i.e., 12-hour fixes). Locations were taken to be as close to the times used in the 12-hour dataset as possible.

We also calculated directedness of migration, also known as the straightness index [51,52], which is defined as the Euclidean distance of migration divided by path distance. Values ranged from 0–1, with lower values indicating a more tortuous migration path and higher values indicating more directed movement. A directedness value of 1 would indicate that the animal migrated in a straight line following the path of least distance during migration.

We compared migration metrics across the coarse and fine wintering areas described in the following section. Because the distribution of many of our metrics (e.g., distance, directedness, and timing of migration) strongly diverged from normality, we evaluated difference of means between wintering areas using a Kruskal-Wallis rank sum test [53], with a multiple comparison test to identify which alternatives differed [54]. We compared duration and directedness of migration overall for all migrants between fall and spring migration using paired Mann-Whitney tests [53].

### Wintering area use and fidelity

Distinct wintering areas were identified using a population-level winter utilization distribution [55–57]. Winter locations were identified for each caribou based on FPT-NSD seasonal breakpoints. For caribou-years for which distinct start and end dates for seasons could not be identified (e.g., residents and other non-migratory movement behaviors), we used the median season start and end dates across all migrant individuals to define the season boundaries. We combined all winter locations from the standardized two-location-per-day dataset and calculated a population-level utilization distribution using kernel density estimation in the R package adehabitatHR [49], using the *ad hoc* approach of Kie [58] to select the optimal bandwidth.

Caribou-years were assigned to the population-level wintering area with which their individual-level utilization distribution had the greatest overlap. We assigned individual-level utilization distributions that did not show any overlap with the various wintering areas to the wintering area to which they were nearest based on the edge-edge distance between the individual’s 95% contour and each wintering area boundary. We analyzed winter use at two scales evident in the winter utilization distribution: coarse use, comparing animals that overwintered on the coastal plain to those that overwintered in or below the Brooks Range, and fine use, comparing use of four individual wintering areas, two each in the coastal plain and Brooks Range.

We defined fidelity as the tendency of an animal to return to its previously used location in consecutive years [59–61]. To analyze the annual fidelity of individual caribou to wintering areas, we calculated transition probabilities using the R package TraMineR [62]. The program created state sequences describing individual-level classifications of coarse- and fine-scale wintering area use and calculated transition probabilities between states. This yielded information on the likelihood of a caribou using the same wintering area in subsequent years or transitioning to a different wintering area. Only individuals with subsequent caribou-years of location data were included in this analysis.

## Results

### Caribou data

Our caribou telemetry dataset consisted of 76 adult female caribou from 2004–2016, with sample size varying per analysis-year (S2 Appendix: S1 Table). Individuals had between 1–8 years of location data (mean 2.2), resulting in 164 caribou-years of data. Thirty-nine caribou (51.3%) had multiple years of data. After collar fix rates were standardized to their coarsest time interval, there were 120 caribou-years with 2-hour fix rates, 17 with 3-hour fix rates, 10 with 8-hour fix rates, and 17 with 12-hour fix rates (73.2%, 10.4%, 6.1%, and 10.4%, respectively).

### Movement classification

The FPT-NSD analysis classified 129/164 (78.7%) annual movement trajectories as migration, 19 (11.6%) as residency, and 16 (9.8%) as some other movement strategy like nomadism. We found interannual variability in the degree of partial migration for the TCH. In any given year between 41–100% of collared caribou migrated (S2 Appendix: S1 Fig).

### Migration characterization

**Fig 2:**
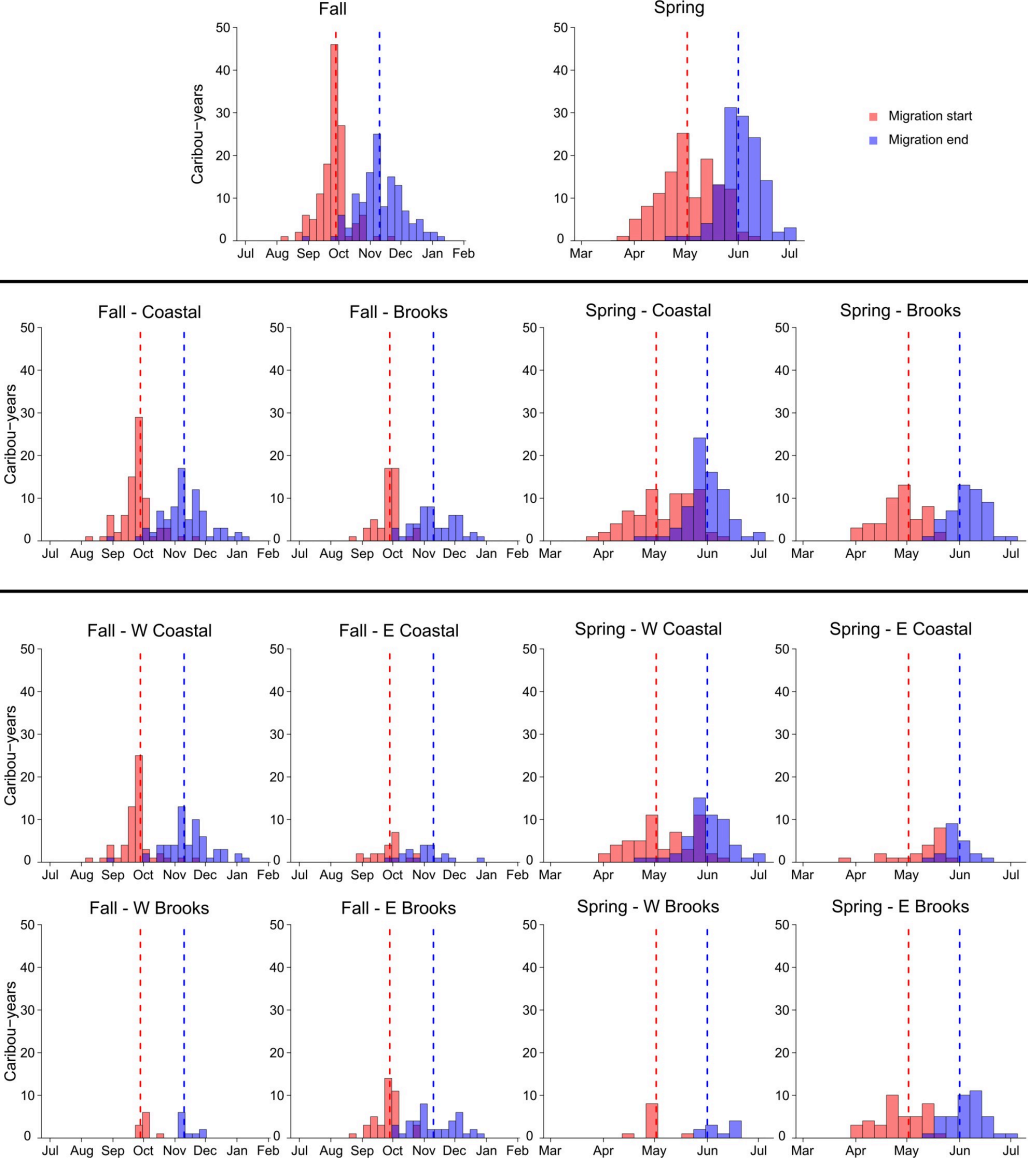
Migration start and end dates by season. Data shown combined for all caribou-years (top row), divided at a coarse scale–coastal plain versus Brooks Range (second row), and divided at a fine scale–four wintering areas (bottom two rows). Bars in each panel represent one-week intervals. Vertical dashed lines indicate median migration start (red) and end (blue) dates, as reported in this study (Table 1). Data are depicted for caribou-years classified as “migration” (n = 129) by the first passage time–net squared displacement method.

Seasonal boundaries identified using median dates across all migrants were similar to those reported by Person et al. [30] for calving and post-calving seasons (Table 1). However, seasonal boundaries differed in other periods. Summer ended almost two weeks later than reported by Person et al. [30], while spring migration started a little over two weeks later but ended at a similar date (Table 1). Fall migration and winter showed opposite patterns, with fall migration starting a week and a half later and ending nearly three weeks earlier than indicated by Person et al. [30], while winter started about two weeks earlier and ended about two weeks later (Table 1). These patterns reflect median season dates, however we observed wide variability in timing across caribou-years (Fig 2).

**Table 1:** Teshekpuk Caribou Herd seasonal boundaries as defined in our study and Person et al. [30].

Source	Summer ^a^	Fall migration	Winter	Spring migration	Calving	Post-calving
This study ^b^	Jul 1 –Sep 27	Sep 28 –Nov 10	Nov 11 –May 1	May 2 –Jun 1	Jun 2 –Jun 16	Jun 17 –Jun 30
Person et al. ^c^	Jul 1 –Sep 15	Sep 16 –Nov 30	Dec 1 –Apr 15	Apr 16 –May 31	Jun 1 –Jun 15	Jun 16 –Jun 30

a. Person et al. [30] split summer into three periods: Mosquito harassment (Jul 1 –Jul 15), mosquito and oestrid fly harassment (Jul 16 –Aug 7), and late summer (Aug 8 – Sep 15). As our focus was on migration and winter, we did not attempt to differentiate summer periods between post-calving and fall migration and instead identified a single summer season.

b. Dates reflect the median start and end dates across all caribou-years classified as migrants (n = 129).

c. Adapted from Russell et al. [63]. See Discussion for details.

**Fig 3:**
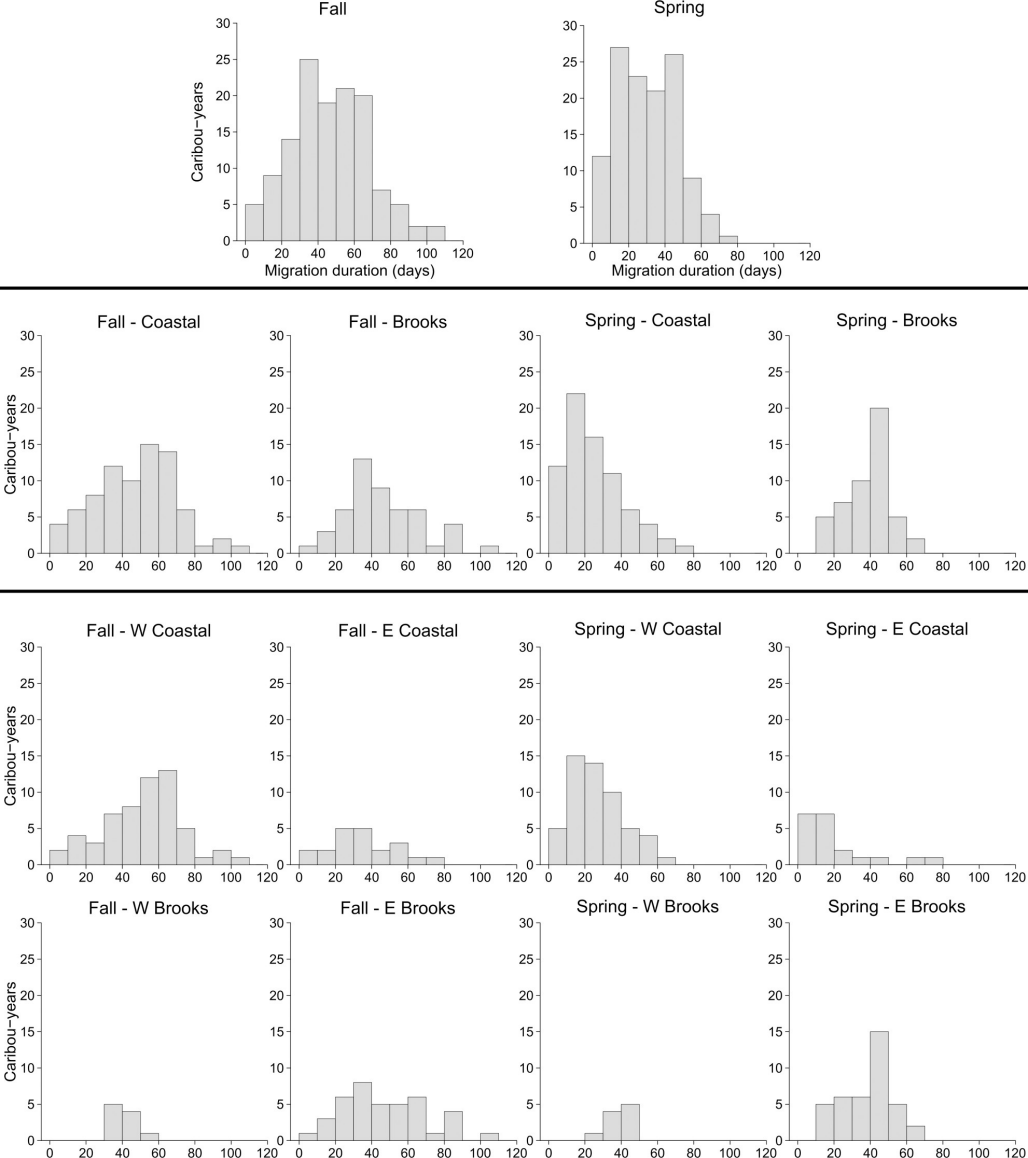
Migration duration (days) by season. Data shown combined for all caribou-years (top row), divided at a coarse scale–coastal plain versus Brooks Range (second row), and divided at a fine scale–four wintering areas (bottom two rows). Data are depicted for caribou-years classified as “migration” (n = 129) by the first passage time–net squared displacement method.

Timing of migration varied by wintering area, with different patterns apparent across seasons (Figs 2 and 3). During fall migration, migrants overwintering on the coastal plain and Brooks Range at a coarse scale did not significantly differ in their start or end dates, nor in their duration of migration (S2 Appendix: S2 Table). At a fine scale, migrants to the four wintering areas ended their migration at a similar time but had different start dates and durations of migration, with the earliest start date and longest duration of migration, on average, for W Coastal migrants (S2 Appendix: S2 Table). In contrast, spring migration tended to exhibit differences at both coarse and fine scales (S2 Appendix: S3 Table). Migrants from the Brooks Range started migration earlier and ended later than those from the coastal plain, resulting in a duration of migration that was about two weeks longer on average (S2 Appendix: S3 Table). Comparing across seasons, duration of migration was significantly longer for fall migration than spring migration (p < 0.001; S2 Appendix: S2 Fig).

**Fig 4:**
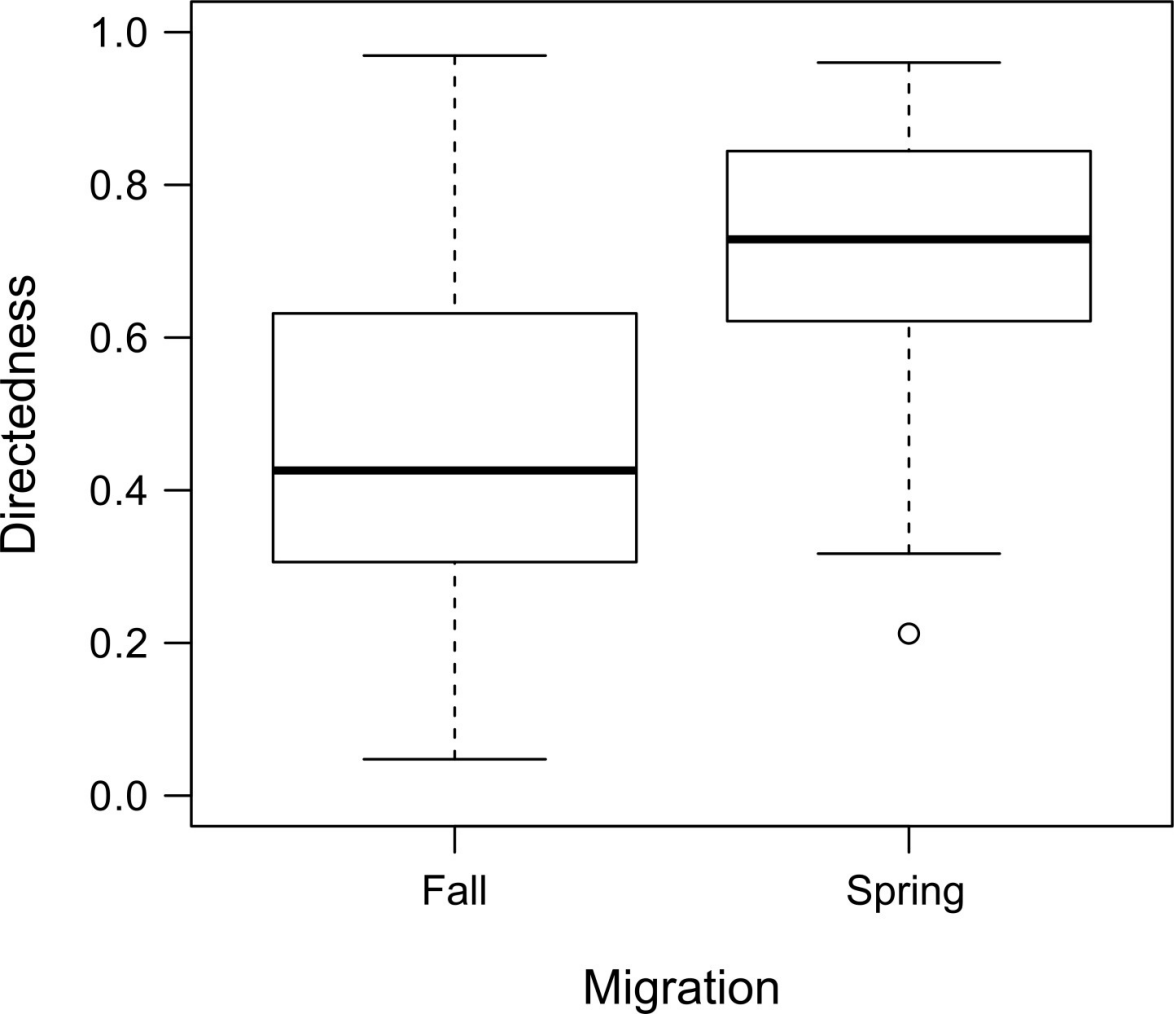
Directedness of fall and spring migration for the Teshekpuk Caribou Herd. Directedness indicates the degree of tortuosity of a caribou’s migration path, with lower values indicating a more tortuous migration path and higher values indicating more directed movement. Across all migrants, spring migration tended to be more directed than fall (p < 0.001).

Migrants to the Brooks Range travelled significantly farther than those that remained on the coastal plain, considering both path distance and Euclidean distance (S2 Appendix: S4 and S5 Tables, S3 and S4 Figs). Comparing overall migration distance across seasons also indicated differences. Fall migration path distances were significantly longer than those during spring migration (p < 0.001), but Euclidean distances did not significantly differ (p = 0.19). This was reflected in the directedness of migration metric, which indicated significantly more directed movement in spring compared to fall (${\bar{x}}_{\mathrm{fall}}$ = 0.46, ${\bar{x}}_{\mathrm{spring}}$ = 0.72, p < 0.001; Fig 4).

### Wintering area use and fidelity

**Fig 5:**
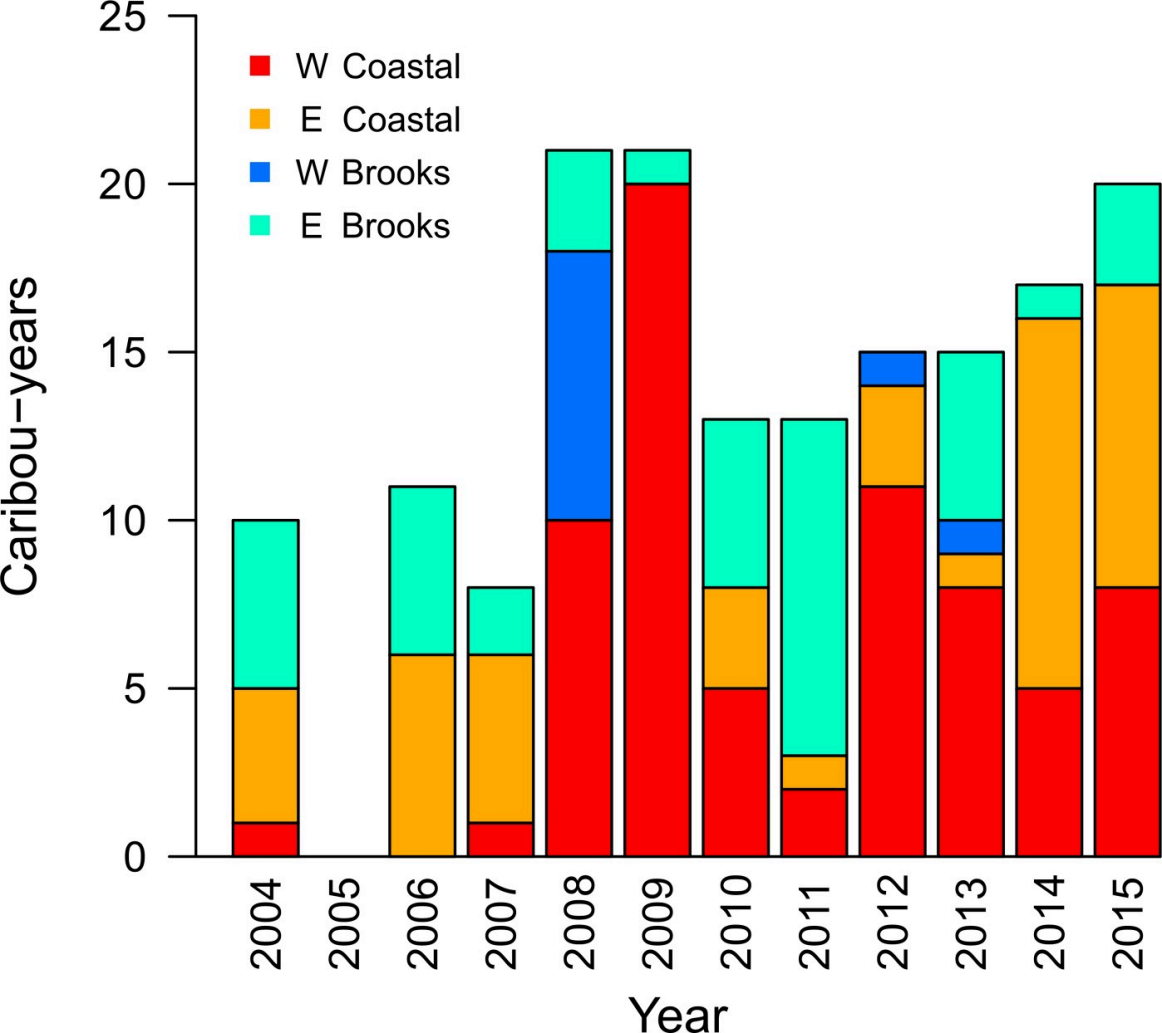
Wintering area use over time for the Teshekpuk Caribou Herd (TCH; n = 164). The x-axis depicts the start of the analysis-year (i.e., 2004 indicates the analysis-year stretching from 1 July 2004–30 June 2005). See Fig 1 for locations of the four wintering areas used by caribou of the TCH.

Four distinct wintering areas were evident in the population-level winter utilization distribution (S2 Appendix: S5 Fig): the western coastal plain (W Coastal), eastern coastal plain (E Coastal), western Brooks Range (W Brooks), and eastern Brooks Range (E Brooks; Fig 1). In the coastal plain region in the north of the study area, where caribou occur at higher densities, we distinguished wintering areas using the 50% contour of the population-level utilization distribution (S2 Appendix: S5 Fig). In the lower-density Brooks Range areas to the south, we distinguished wintering areas using the 85% contour (S2 Appendix: S5 Fig). Most caribou-years showed winter overlap with at least one of the four wintering areas. Six caribou had individual-level utilization distributions that did not show overlap with any wintering area. Based on the edge-edge distance between the individual’s 95% contour and each wintering area contour, one was assigned to the W Brooks, three to the E Brooks and two to the E Coastal. This resulted in a total of 71 caribou-years showing overwintering in the W Coastal area (43.3%), 43 in the E Coastal (26.2%), 10 in the W Brooks (6.1%), and 40 in the E Brooks (24.4%). Use of wintering areas varied over time (Fig 5). Notably, however, multiple wintering areas were used each year. Furthermore, each wintering area had at least one analysis-year in which it was not used by collared caribou.

State sequence analyses of the 39 caribou (51.3%) with multiple caribou-years of location data (78 total subsequent-year transitions), revealed differences in the degree of wintering area fidelity at coarse and fine scales of wintering area use. At a coarse scale, comparing use of the coastal plain versus Brooks Range, the probability of wintering on the coastal plain was largely independent of where a caribou wintered the previous year (Table 2). Individuals that overwintered on the coastal plain in one year were highly likely to winter on the coastal plain the following year, while those that overwintered in the Brooks Range were likely to switch to the coastal plain the following year (Table 2). This is consistent with observed patterns of heavier use of the coastal plain in general, despite annual variability (Fig 5) [30]. At a fine scale, fidelity to the four individual wintering areas was low for most areas (Table 3). Probabilities of individual caribou overwintering in the same fine-scale wintering area in subsequent years were less than 0.35 for all wintering areas except for the W Coastal, which showed a slightly higher likelihood of reuse (prob. = 0.57).

**Table 2:** Transition probabilities for caribou use of the coastal plain in the north of the study area (Coastal) and the Brooks Range mountains in the south of the study area (Brooks) in subsequent winters.

	Coastal (n = 58)	Brooks (n = 20)
Coastal (n = 51)	0.76	0.24
Brooks (n = 27)	0.70	0.30

Probabilities are rounded to the second decimal place and indicate the likelihood of an individual caribou moving from the row location in one year to the column location in the next year. Sample sizes indicate the number of caribou starting in (rows) or ending in (columns) each wintering area for the 78 observed subsequent-year transitions.

**Table 3:** Transition probabilities for caribou moving between fine-scale wintering areas in subsequent years.

	W Coastal (n = 40)	E Coastal (n = 18)	W Brooks (n = 2)	E Brooks (n = 18)
W Coastal (n = 37)	0.57	0.22	0.00	0.22
E Coastal (n = 14)	0.43	0.29	0.00	0.29
W Brooks (n = 8)	0.88	0.12	0.00	0.00
E Brooks (n = 19)	0.32	0.26	0.11	0.32

See Fig 1 for wintering area locations. Probabilities are rounded to the second decimal place and indicate the likelihood of an individual caribou moving from the row location in one year to the column location in the next year. Sample sizes indicate the number of caribou starting in (rows) or ending in (columns) each wintering area for the 78 observed subsequent-year transitions.

## Discussion

Migratory species depend on access to seasonal ranges to meet the myriad ecological pressures faced by individual animals [4]. We investigated migration flexibility in a long-distance arctic migrant by classifying movement behavior, characterizing migration dynamics, and analyzing wintering area fidelity for the partially migratory TCH. Migration was a dominant strategy in most years for the TCH (S2 Appendix: S1 Fig), as has been noted for other large caribou herds in Alaska [64]. This is emblematic of a wider trend among many ungulates worldwide in which migrants tend to be much more abundant than residents in populations due to benefits of improved nutrition and reduced predation/disease [65,66] (but see [12,67]). Among Alaskan caribou, herds with long distance migrations tend to be much larger than herds with more localized, year-round distributions [40]. Aside from the TCH, the proportion of migrants within caribou herds has typically not been reported (but see [64]) and is an area that warrants future research, including tracking over time as it may vary annually (S2 Appendix: S1 Fig).

### Challenges in classifying migratory movement

While most caribou of the TCH were identified as migrants, we documented a wide array of movement variability within what we classified as migration. Within the migrants, there was a spectrum of migratory distances and durations observed (Fig 3, S2 Appendix: S3 and S4 Figs). This variability likely contributed to the challenges that several previously published techniques had classifying movement behavior for the TCH (S1 Appendix). There were also multiple individuals that, even though classified as residents or other movement types, nonetheless showed “migration-like” movements where periods of increased movement rate and more directed movement started around the same time as migration for other individuals, even though the end results were not clear enough to classify as migration. Similarly, Nicholson et al. [64] noted that there were some caribou in their study of the Central Arctic Herd that did not fit their strict definition of migration but nonetheless showed movements between distinct ranges across seasons. Within migratory species, some environmental or endogenous cues may result in migratory restlessness, even when a migration does not actually take place [68]. These observations suggest that greater attention is needed to clarify what constitutes migratory movements, depending upon the ecological questions at hand.

We recorded a hierarchy of movement, including coarse- and fine-scale wintering area use. By some definitions, all individuals that overwintered on the coastal plain would be considered residents and only those that moved to the Brooks Range would be counted as migrants. However, this would mask a great deal of diversity in movement behaviors and patterns. Movement differences can have fitness consequences for ungulates and should not simply be ignored. For example, mule deer (*Odocoileus hemionus*) that migrate longer distances spend significantly more time migrating and have greater exposure to potential barriers like highways and fences, but exhibit reduced risk of human harvest compared to shorter distance migrants [11]. As studies of migratory species continue to increase worldwide, we recommend increased attention to the variability of behaviors within what is considered migration.

### Caribou migration dynamics

Many ungulate migratory behaviors are flexible, responding to fluctuating environmental conditions with variable routes and timing [7]. Nonetheless, patterns have emerged such as migration distance influencing timing with longer distance migrants spending more time migrating [11]. We found this pattern to be supported at a coarse scale for TCH spring migration, as migrants from the Brooks Range–which travelled farther on average than coastal plain migrants (S2 Appendix: S4 and S5 Tables)–left earlier and arrived later, resulting in a significantly longer duration of migration (S2 Appendix: S3 Table). During fall migration, however, distances differed significantly (S2 Appendix: S4 and S5 Tables) but there were not significant differences in departure, arrival, or duration of migration (S2 Appendix: S2 Table).

Our finding of more directed movement in spring than in fall (Fig 4) contrasts with patterns reported for the Central Arctic Herd [64,69], though it is not clear how directedness was determined in those studies. Nicholson et al. [64] suggested less directed movement in spring may be due to poor body condition and the high energy demands of gestation limiting the ability to travel rapidly in spring. However, a study of the nearby Western Arctic Herd found that fall migration more closely aligned with a random walk movement model than a more linear least cost path model [70], suggesting less directed movement in the fall. This may reflect a greater degree of exploratory movement to enhance foraging opportunities prior to winter that is enabled by milder, prolonged fall seasons and necessitated by the lack of individual fidelity to wintering areas. In contrast, straighter paths might be expected during spring migration when movements are toward calving grounds that are well known to the caribou [71,72] and long established as an area with high fidelity [41,73,74]. Further studies are needed to confirm if these hypotheses explaining migratory directedness in fall and spring are supported. Such inquiry will benefit from a replicable and comparable means of quantifying directedness of migration. Our use of the ratio of Euclidean to path distance presents one such metric and we encourage its continued use in comparative studies of caribou and other species to further understanding of migration behavior across seasons.

Migration and winter dates used by Person et al. [30] differed from those we identified by 2–3 weeks (Table 1). The dates in Person et al. [30] were modified from seasonal dates reported by Russell et al. [63] for the Porcupine Caribou Herd. Russell et al. [63] chose dates that reflected changes in environmental conditions that may have ramifications for caribou energy use and behavior, whereas our dates are movement-driven, based on observed differences in movement behavior. Our dates are more similar to those reported for the Central Arctic Herd [64], which were also based on movement data. These patterns reinforce the importance of careful consideration of choice of seasonal boundary dates as indicators of movement behavior.

### Wintering area fidelity and migration plasticity

Despite the predominance of migratory behavior in the TCH, we identified substantial variability in use of wintering areas over time. We observed use of four distinct wintering areas, with low individual-level fidelity reflected in the generally low probability of an individual TCH caribou reusing a given wintering area in subsequent years. This aligns with a recent comparative study that found caribou had the lowest overall site fidelity of the 8 ungulate species investigated [60]. Patterns of spatial fidelity appear to vary throughout the year for caribou, however, with the nearby Western Arctic Herd exhibiting strong fidelity for their general calving area, though with variation in annual calving areas, and weakest fidelity in winter [71,74].

Although individual-level winter fidelity appears to be low, there seems to be overall consistency in herd-level patterns of winter use over two and a half decades (1990–2015). Person et al. [30] indicated comparable use of the coastal plain (65% of individuals using the combined areas of the coastal plain in Person et al. compared to 70% in this study), E Brooks (21% versus 24%), and W Brooks (11% versus 6%) by TCH caribou during winter. This observation of strong herd-level winter fidelity, even during large changes in herd size [75], is notable in comparison with adjacent herds that have demonstrated a more decadal pattern of using a given wintering area, followed by near or total abandonment [76,77]. The difference may lay in the density of caribou during winter, with higher cumulative winter densities of caribou across years for other herds increasing the likelihood of abandonment. This warrants further investigation, along with additional research to reveal what factors drive winter range selection and long-term patterns in use, as well as any population-level demographic implications for caribou.

Weak year-to-year individual fidelity and behavioral flexibility may provide benefits to species [60,78], especially in situations of increasing environmental variability such as with climate change [79–81], or when seasonal ranges become overutilized and exploitation of new habitats can be beneficial [82]. However, these benefits may apply differently across wintering areas, based on differences in wintering area conditions. Predator density [83–86], forage quantity or quality [87–89], snow depth and density [90,91], or exposure to human development and activity [92] may influence TCH use of specific wintering areas. Individual factors such as body size, presence of a calf, or age may compound these differences, leading to varying nutritional needs, sensitivity to disturbance, and susceptibility to predation, parasites, or disease. These sources of variability make it likely that potential fitness tradeoffs will differ among individuals and years. For example, migrants and residents may vary in their body condition, demographic performance, and exposure to threats [11,93,94]. Such fitness tradeoffs may also extend beyond the winter period, leading to seasonal carryover effects in which environmental conditions in one place or season lead to differences among individuals or populations that affect demographic rates in subsequent locations and seasons [7,95,96]. This reinforces the importance of understanding the ecological processes that drive within- and between-individual variation in migration and winter fidelity observed in this study.

### Conservation and management implications

Our study adds caribou to the growing list of ungulates that exhibit individual-level migratory plasticity [18,19,97–101]. New efforts to map ungulate migrations seek to increase awareness of the threats to ungulate migration and to provide data to support their conservation and management [102]. Understanding dynamics of migratory behavior, destination, and timing plays an important role in supporting such goals. As these efforts proceed, it is important that they reflect migratory variability not only between, but also within, populations. While the importance of conserving biodiversity is well recognized, much attention is focused at the species level or higher. Within-species diversity may play key roles in providing ecosystem services that benefit both people and nature [103] and can have ecological effects as great as, or even greater than, species-level effects [104]. This underscores the value of attention to within-species diversity in future conservation efforts.

Given the recognized consequences of disrupted migration for large herbivore populations [7], it is important for land use decisions to account for potential impacts to migratory connectivity. Recent decisions have expanded the amount of TCH habitat available for oil and gas leasing and development [105] and approved new projects across the range of the herd (e.g., [36,106]). Understanding patterns of winter use and migration in areas proposed for development may allow analysis of potential impacts of proposed projects [107] as well as influence site selection and mitigation decisions. It is unknown how overwintering caribou in northern Alaska will respond to infrastructure and human activities, as most North Slope development to date has occurred outside of primary caribou winter range. Studies in Canada, however, have found avoidance of infrastructure by overwintering caribou [92,108]. With the TCH making heavy use of the coastal plain during winter, there is a need to similarly investigate patterns of winter response to infrastructure and to inform management and permitting decisions accordingly. There is also a need for better understanding the effects on caribou of temporary infrastructure such as ice and snow roads, exploratory drilling, and winter seismic exploration.

Improved understanding of movement dynamics is not just relevant for caribou and the species that they influence, but also for humans who rely on caribou. Annual movements of the TCH bring them in proximity to several northern Alaskan communities (Fig 1) populated primarily by Alaska Native peoples. Subsistence hunting for caribou and other species is crucial for food security in these remote communities that are not connected to permanent infrastructure and so have high costs to import food [109]. Harvest also is an important part of the culture, identity, and customary and traditional ways of life for people in the region [109,110]. There is increasing recognition that loss of caribou and their migrations can convey considerable emotional and cultural toll on Indigenous people [111,112]. Thus, understanding natural variation in timing and destination of caribou migration, as well as how migration is affected by climate change and human activity, has direct impacts for Indigenous culture and for subsistence management.

## Conclusions

We found within-individual variation in movement behavior and wintering area use by the TCH, adding caribou to the growing list of ungulates that exhibit migratory plasticity. Recent work has emphasized that expression of migration can be state, condition, or density dependent [13,113–115]. Understanding drivers of migration at both proximate and ultimate levels is key to understanding how they will be affected by changing environmental conditions [116]. As caribou face the pressures of a rapidly warming Arctic [117] and ongoing human development and activities [107,118,119], further research is needed to investigate what factors influence this diversity of caribou behaviors in Alaska and across the circumpolar Arctic.

## Supporting information

S1 Appendix Comparison of movement classification approaches. S1 Fig. Population-level mean variance (solid black line) ± SE (dashed black lines) of log-transformed first passage time (FPT) as a function of radius. Variance is maximized at a radius of 9 km, indicating that this is the range at which the Teshekpuk Caribou Herd individuals in this study perceive their environment.
(PDF)

S2 Appendix Additional tables and figures. S1 Table. Sample size of movement data per analysis-year for the Teshekpuk Caribou Herd in northwestern Alaska. Analysis-years begin 1 July of the indicated year and continue through 30 June of the following year. All caribou had their GPS collars replaced with Platform Terminal Transmitter (PTT) collars in 2005, resulting in a lack of GPS records for the 2005 analysis-year. S2 Table. Timing of fall migration for the Teshekpuk Caribou Herd. Start date, end date, and duration of migration are reported at three scales: Overall, by coarse wintering area, and by fine wintering area. Values with different superscript letters had statistically significant (p < 0.05) differences in timing. Comparisons were only done within each timing metric and scale, not between metrics or scales. S3 Table. Timing of spring migration for the Teshekpuk Caribou Herd. Start date, end date, and duration of migration are reported at three scales: Overall, by coarse wintering area, and by fine wintering area. Values with different superscript letters had statistically significant (p < 0.05) differences in timing. Comparisons were only done within each timing metric and scale, not between metrics or scales. S4 Table. Path distances (km) for Teshekpuk Caribou Herd fall and spring migration. Path distance was calculated for caribou-years classified as “migration” (n = 129) by summing the distances between consecutive locations during an individual’s migration period (as determined by the first passage time–net squared displacement method) on datasets standardized to have at most two locations per day. Patterns were similar for Euclidean distance (S2 Appendix: S5 Table). Distances are reported at three scales: Overall, by coarse wintering area, and by fine wintering area. Values with different superscript letters had statistically significant (p < 0.05) differences in migration distance. Comparisons were only done within each season and scale, not between scales. S5 Table. Euclidean distances (km) for Teshekpuk Caribou Herd fall and spring migration. Values report the Euclidean distance between individual migration start and end locations (as determined by the first passage time–net squared displacement method), using datasets standardized to have at most two locations per day, for caribou-years classified as “migration” (n = 129). Distances are reported at three scales: Overall, by coarse wintering area, and by fine wintering area. Values with different superscript letters had statistically significant (p < 0.05) differences in migration distance. Comparisons were only done within each season and scale, not between scales. S1 Fig. Percentage of caribou classified as migrants in each analysis-year by the first passage time–net squared displacement (FPT-NSD) analysis. Analysis-years begin 1 July of the indicated year and continue through 30 June of the following year. Confidence intervals were calculated using the Wilson method from the binom R package. S2 Fig. Duration of fall and spring migration for the Teshekpuk Caribou Herd. Across all migrants (n = 129), fall migration tended to take longer than spring migration (p < 0.001). S3 Fig. Migration path distance (km) by season. Data shown combined for all caribou-years (top row), divided at a coarse scale–coastal plain versus Brooks Range (second row), and divided at a fine scale–four wintering areas (bottom two rows). Path distance was calculated for caribou-years classified as “migration” (n = 129) by summing the distances between consecutive locations during an individual’s migration period (as determined by the first passage time–net squared displacement method) on datasets standardized to have at most two locations per day. See S2 Appendix: S4 Fig for results using Euclidean distance of migration. S4 Fig. Euclidean distance of migration (km) by season. Data shown combined for all caribou-years (top row), divided at a coarse scale–coastal plain versus Brooks Range (second row), and divided at a fine scale–four wintering areas (bottom two rows). Data are depicted for caribou-years classified as “migration” (n = 129) by the first passage time–net squared displacement method. See S2 Appendix: S3 Fig for path distance of migration. S5 Fig. Winter population-level utilization distribution for the Teshekpuk Caribou Herd. We used the 50% contours (solid black lines) to distinguish wintering areas in the high-density coastal plain region and 85% contours (dashed black lines) in the lower density Brooks Range region. See Fig 1 for a depiction of final wintering areas.
(PDF)

S3 Appendix Data. S1 Metadata. Description of the data files contained in S3 Appendix. S1 Data. Caribou movement data summary file. Summary.csv file containing data for the 164 caribou-years of Teshekpuk Caribou Herd movement data analyzed in this study. S2 Data. TCH winter population-level utilization distribution raster. Raster file in GeoTiff format depicting the population-level winter utilization distribution for the Teshekpuk Caribou Herd, as shown in S2 Appendix: S5 Fig.
(ZIP)
