# Supplementary material for: Variation in winter site fidelity within and among individuals influences movement behavior in a partially migratory ungulate
Source: PLoS One. 2021 Sep 30;16(9):e0258128. doi: 10.1371/journal.pone.0258128 (PMC8483381; doi:10.1371/journal.pone.0258128)
Supplement: S1 Appendix — S1 Fig. Population-level mean variance (solid black line) ± SE (dashed black lines) of log-transformed first passage time (FPT) as a function of radius. Variance is maximized at a radius of 9 km, indicating that this is the range at which the Teshekpuk Caribou Herd individuals in this study perceive their environment. (PDF) [file pone.0258128.s001.pdf]

Fullman, T.J., Person, B.T., Prichard, A.K., Parrett, L.S. 2021. Variation in winter site fidelity within and among individuals influences movement behavior in a partially migratory ungulate. PLOS ONE.

S1 Appendix: Comparison of movement classification approaches

We evaluated four potential methods for classifying movement behavior of caribou: home range overlap [39], latent state modeling of net squared displacement [42], mechanistic range shift analysis [18], and first-passage time – net squared displacement [43,44].

Overview of the four methods

Home range overlap [39] attempts to identify seasonal habitat use by analyzing animal location data across various time windows (e.g., Jul-Nov, Dec-Mar, Apr-Jun). Location data from a single analysis-year are divided into two or three groups and kernel density estimation is used to define a utilization distribution for the individual's use in each period. The overlap between pairs of ranges is then calculated. This is repeated for all possible combinations of two-season or three-season ranges and the combination that yields the minimum overlap for successive ranges is chosen as that which can be used to determine whether an animal migrated. If the level of overlap is greater than the median level of minimum overlap across the population the animal is classified as a resident, otherwise it is evaluated for migration or "no-return" movement. For further details, see Cagnacci et al. [39].

Latent state modeling of net squared displacement (LSM NSD) is an extension of the net squared displacement approach used in many animal migration studies [42]. It applies a discrete latent state model to NSD data to overcome issues with failed model convergence, poor fit, and

temporal autocorrelation. At every time step along an individual's location time series, the NSD value is associated with a movement mode, represented using a mixture model with two normal distributions (reflecting encamped movements, e.g. winter use, calving) and one uniform distribution (reflecting exploratory movement, e.g. travel). Time steps are assigned to one of the three movement modes using a Bayesian Monte Carlo Markov-Chain approach and a transition probability between models is calculated. Based on the transition probabilities and the number of observed transitions, Bastille-Rousseau et al. [42] provide rules to assign movement behavior to individuals.

The mechanistic range shift analysis (MRSA) provides an approach in which various movement parameters are estimated while accounting for common issues in telemetry data, such as autocorrelation and irregular sampling intervals [18]. Multiple possible movement models are fit to the location data using likelihood methods and compared using the Akaike Information Criterion (AIC). The model has options for significance tests of various phenomenon, such as whether a range shift occurred, whether there was a stopover in the movement data, and whether the animal returned to its previous year's seasonal range.

The first passage time – net squared displacement approach (FPT-NSD) combines movement-based and location-based data to identify seasonal movement patterns [43,44]. First passage time (FPT) describes the time required by an animal to pass through a circle of a given radius, with higher FPT values denoting more tortuous movement while lower FPT values denote faster, more linear movement [45]. To determine the appropriate radius for calculating FPT, the metric is calculated on the annual trajectories of all collared individuals using multiple radii. The species is considered to perceive its environment at the radius at which the variance of log(FPT) is maximized [45]. We tested radii ranging from 1 km to 300 km (radius increment:

1-15 km every km, 20-100 km every 5 km, 110-300 km every 10 km). Taking the mean across all collared individuals, we found the maximum variance and used that radius to calculate FPT for each individual. The mean variance of log(FPT) across all collared individuals was maximized at r = 9 km (S1 Fig). Following Fauchald and Tveraa [45], we consider this the scale at which the TCH perceives its environment on an average annual basis.

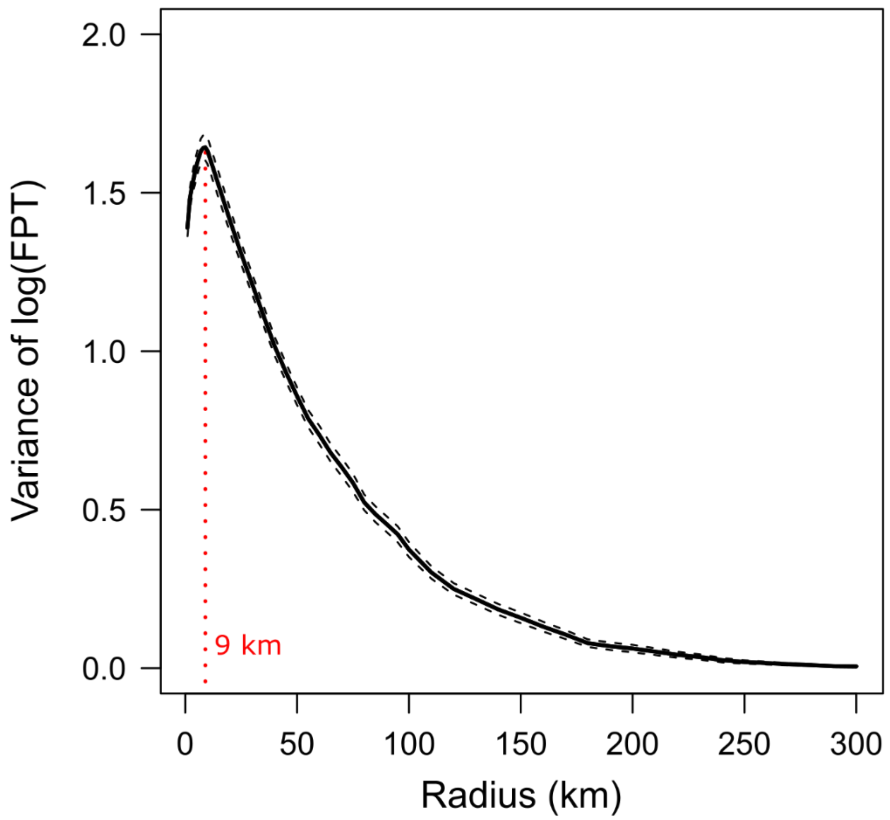

S1 Fig. Population-level mean variance (solid black line) ± SE (dashed black lines) of log-transformed first passage time (FPT) as a function of radius. Variance is maximized at a radius of 9 km, indicating that this is the range at which the Teshekpuk Caribou Herd individuals in this study perceive their environment.

We subdivided the movement paths for each caribou-year into groups with similar FPT values using a segmentation process [46] to indicate breakpoints in movement behavior. Large differences in FPT values can swamp the ability of the segmentation procedure to detect finer seasonal changes so a first segmentation was run to identify mid-winter locations (high clustering with corresponding high FPT values) and then additional segmentations were run on fall and spring segments to distinguish migration from summer, calving, and other movement periods [44]. The segmentation process often indicated more breakpoints than just those surrounding migration (e.g., the summer period, prior to fall migration, might be subdivided into three segments: high movement to reach insect relief habitat, clustering during insect relief, and high movement post-insect relief but before migration). We manually reviewed candidate FPT breakpoints for each individual to classify seasonal movement periods. Net squared displacement (NSD) values played an important role in this review. Commonly used in animal movement studies, NSD reports straight line distances between initial and subsequent values and are used to signal changes in movement areas [47]. We used two sets of NSD values. The first reflected the distance of subsequent locations from the first recorded location of each analysis-year. The second set of NSD values indicated the distance of each location from the first day of winter behavior [44]. In some situations, relating this second NSD curve to the FPT breakpoints provided additional clarity as to which reflected season boundaries. We used a combination of FPT values, NSD values, and visual analysis of segmented locations to assign locations to different seasons and identify seasonal boundaries. For additional details see the main text.

Comparison results

In comparing the four methods for classifying animal movement, only FPT-NSD proved suitable for discrimination of seasonal movement behaviors of the Teshekpuk Caribou Herd (TCH). For this herd, variable patterns of clustering and movement throughout the year meant that the home range overlap technique often identified minimum overlap for a single month of tightly clustered locations. This resulted in the assigned groups before or after this month including migration along with summer or calving periods, and sometimes part of winter, leading to erroneous estimates of migration and timing. While refinements such as inclusion of time constraints on the windows of analysis may improve upon the classification ability of this approach, we did not pursue these and did not continue to use the home range overlap approach in our analysis. The LSM NSD technique was effective for classical migrants with nicely characterized NSD curves, similar to the ideal depicted in Bunnefeld et al. [47]. Many caribou in our data set, however, did not show tight summer and winter clusters with fairly linear movements between them and so misclassifications were common, preventing further use of this approach. The MRSA approach presents an intriguing option for analyzing animal movement data that has great potential. At the time of our analysis, however, model constraints such as assuming the size of the area across which animals range is the same across seasons (whereas in caribou the summer range typically is vastly larger than the winter range for a given individual) led us to find the model unsuitable for our purposes.

Conclusion

Discriminating movement behavior appears to be strongly influenced by the movement variability exhibited by a particular species and by individuals within a population. It is further complicated by complexity in the question of what qualifies as migratory behavior, which has long been discussed in the animal movement literature [4]. This issue featured strongly in our comparison of movement classification techniques and in influencing which approaches were suitable for the TCH. Many of the approaches we investigated appear to be best suited for identifying migration in species that exhibit stereotypical migrant behavior – moving widely during the migratory period and using concentrated areas during the other periods. Barren ground caribou on the North Slope of Alaska, however, diverge from this pattern, featuring some of their highest movement rates during the summer insect harassment period [30,37,38], prior to fall migration. Previous work has noted the difficulty in using quantitative approaches to reliably distinguish migration due to variability in caribou movement patterns [64]. Even during the winter, when caribou are typically thought to be sedentary, we observed some individuals that would cluster in one location for a time and then suddenly move to a new area of winter clustering. While such within-season movements have been noted for other species (e.g., [120]), further research is needed to understand what drives these sudden winter movements for caribou.

Additional tools are needed that classify migratory movements in a flexible manner to account for the variability in migration behavior and annual movement patterns of highly mobile species like caribou. We found that the FPT-NSD approach was useful for discriminating movement behavior, albeit with a substantial amount of researcher investment. The FPT-NSD approach presents a step forward in allowing classification that provides quantitative measures of some of the more qualitative metrics used in previous studies (e.g., [64]), but is still a labor-

intensive process that relies on some subjectivity in determining which breakpoints indicate season transitions. Promising tools exist that seek to robustly classify movement behavior (e.g., [18]) and we encourage their continued development to account for a wider diversity of what constitutes migratory movement in highly mobile species.

References

4. Dingle H, Drake VA. What Is Migration? Bioscience. 2007;57: 113–121.

18. Gurarie E, Cagnacci F, Peters W, Fleming CH, Calabrese JM, Mueller T, et al. A framework for modelling range shifts and migrations: asking when, whither, whether and will it return. J Anim Ecol. 2017;86: 943–959. doi:10.1111/1365-2656.12674

30. Person BT, Prichard AK, Carroll GM, Yokel DA, Suydam RS, George JC. Distribution and movements of the Teshekpuk Caribou Herd 1990-2005: Prior to oil and gas development. Arctic. 2007;60: 238–250.

37. Fancy SG, Pank LF, Whitten KR, Regelin WL. Seasonal movements of caribou in artic Alaska as determined by satellite. Can J Zool. 1989;67: 644–650. doi:10.1139/z89-093

38. Prichard AK, Yokel DA, Rea CL, Person BT, Parrett LS. The effect of frequency of telemetry locations on movement-rate calculations in arctic caribou. Wildl Soc Bull. 2014;38: 78–88. doi:10.1002/wsb.357

39. Cagnacci F, Focardi S, Ghisla A, van Moorter B, Merrill EH, Gurarie E, et al. How many routes lead to migration? Comparison of methods to assess and characterize migratory movements. J Anim Ecol. 2016;85: 54–68. doi:10.1111/1365-2656.12449

42. Bastille-Rousseau G, Potts JR, Yackulic CB, Frair JL, Ellington EH, Blake S. Flexible characterization of animal movement pattern using net squared displacement and a latent state model. Mov Ecol. 2016;4: 15. doi:10.1186/s40462-016-0080-y

43. Le Corre M, Dussault C, Côté SD. Detecting changes in the annual movements of terrestrial migratory species: using the first-passage time to document the spring migration of caribou. Mov Ecol. 2014;2: 19. doi:10.1186/s40462-014-0019-0

44. Le Corre M, Dussault C, Côté SD. Weather conditions and variation in timing of spring and fall migrations of migratory caribou. J Mammal. 2017;98: 260–271. doi:10.1093/jmammal/gyw177

45. Fauchald P, Tveraa T. Using First-Passage Time in the analysis of area-restricted search and habitat selection. Ecology. 2003;84: 282–288. doi:10.1890/0012-9658(2003)084[0282:UFPTIT]2.0.CO;2

46. Lavielle M. Using penalized contrasts for the change-point problem. Signal Processing. 2005;85: 1501–1510. doi:10.1016/j.sigpro.2005.01.012

47. Bunnefeld N, Börger L, van Moorter B, Rolandsen CM, Dettki H, Solberg EJ, et al. A model-driven approach to quantify migration patterns: individual, regional and yearly differences. J Anim Ecol. 2011;80: 466–476. doi:10.1111/j.1365-2656.2010.01776.x

64. Nicholson KL, Arthur SM, Horne JS, Garton EO, Del Vecchio PA. Modeling caribou movements: Seasonal ranges and migration routes of the Central Arctic Herd. PLOS ONE. 2016;11: e0150333. doi:10.1371/journal.pone.0150333

120. Couriot O, Hewison AJM, Saïd S, Cagnacci F, Chamaillé-Jammes S, Linnell JDC, et al. Truly sedentary? The multi-range tactic as a response to resource heterogeneity and unpredictability in a large herbivore. Oecologia. 2018;187: 47–60.
