# Supplementary material for: Variation in winter site fidelity within and among individuals influences movement behavior in a partially migratory ungulate
Source: PLoS One. 2021 Sep 30;16(9):e0258128. doi: 10.1371/journal.pone.0258128 (PMC8483381; doi:10.1371/journal.pone.0258128)
Supplement: S2 Appendix — S1 Table. Sample size of movement data per analysis-year for the Teshekpuk Caribou Herd in northwestern Alaska. Analysis-years begin 1 July of the indicated year and continue through 30 June of the following year. All caribou had their GPS collars replaced with Platform Terminal Transmitter (PTT) collars in 2005, resulting in a lack of GPS records for the 2005 analysis-year. S2 Table. Timing of fall migration for the Teshekpuk Caribou Herd. Start date, end date, and duration of migration are reported at three scales: Overall, by coarse wintering area, and by fine wintering area. Values with different superscript letters had statistically significant (p < 0.05) differences in timing. Comparisons were only done within each timing metric and scale, not between metrics or scales. S3 Table. Timing of spring migration for the Teshekpuk Caribou Herd. Start date, end date, and duration of migration are reported at three scales: Overall, by coarse wintering area, and by fine wintering area. Values with different superscript letters had statistically significant (p < 0.05) differences in timing. Comparisons were only done within each timing metric and scale, not between metrics or scales. S4 Table. Path distances (km) for Teshekpuk Caribou Herd fall and spring migration. Path distance was calculated for caribou-years classified as “migration” (n = 129) by summing the distances between consecutive locations during an individual’s migration period (as determined by the first passage time–net squared displacement method) on datasets standardized to have at most two locations per day. Patterns were similar for Euclidean distance (S2 Appendix: S5 Table). Distances are reported at three scales: Overall, by coarse wintering area, and by fine wintering area. Values with different superscript letters had statistically significant (p < 0.05) differences in migration distance. Comparisons were only done within each season and scale, not between scales. S5 Table. Euclidean distances (km) [file pone.0258128.s002.pdf]

Fullman, T.J., Person, B.T., Prichard, A.K., Parrett, L.S. 2021. Variation in winter site fidelity within and among individuals influences movement behavior in a partially migratory ungulate. PLOS ONE.

S2 Appendix: Additional tables and figures

S1 Table. Sample size of movement data per analysis-year for the Teshekpuk Caribou Herd in northwestern Alaska.

Year	2004	2005	2006	2007	2008	2009	2010	2011	2012	2013	2014	2015
n	10	0	11	8	21	21	13	13	15	15	17	20
%	6.1	--	6.7	4.9	12.8	12.8	7.9	7.9	9.1	9.1	10.4	12.2

Analysis-years begin 1 July of the indicated year and continue through 30 June of the following year. All caribou had their GPS collars replaced with Platform Terminal Transmitter (PTT) collars in 2005, resulting in a lack of GPS records for the 2005 analysis-year.

S2 Table. Timing of fall migration for the Teshekpuk Caribou Herd.

	Start date			End date			Duration (days)			
	$\bar{x}$	min	max	$\bar{x}$	min	max	$\bar{x}$	sd	min	max
Overall	Sep 26	Aug 11	Nov 19	Nov 12	Aug 30	Jan 12	47	21	5	106
Coastal	Sep 25 [a]	Aug 11	Nov 19	Nov 12 [e]	Aug 30	Jan 12	47 [g]	21	5	104
Brooks	Sep 28 [a]	Aug 24	Oct 24	Nov 12 [e]	Oct 3	Dec 24	46 [g]	21	9	106
W Coastal	Sep 25 [c]	Aug 11	Nov 19	Nov 15 [f]	Aug 30	Jan 12	52 [h]	21	7	104
E Coastal	Sep 27 [cd]	Sep 1	Oct 26	Nov 2 [f]	Sep 27	Dec 30	35 [i]	18	5	72
W Brooks	Oct 4 [d]	Sep 26	Oct 18	Nov 14 [f]	Nov 6	Nov 30	40 [i]	6	32	52
E Brooks	Sep 26 [cd]	Aug 24	Oct 24	Nov 12 [f]	Oct 3	Dec 24	47 [hi]	23	9	106

Start date, end date, and duration of migration are reported at three scales: overall, by coarse wintering area, and by fine wintering area. Values with different superscript letters had statistically significant ($p < 0.05$) differences in timing. Comparisons were only done within each timing metric and scale, not between metrics or scales.

S3 Table. Timing of spring migration for the Teshekpuk Caribou Herd.

	Start date			End date			Duration (days)			
	$\bar{x}$	min	max	$\bar{x}$	min	max	$\bar{x}$	sd	min	max
Overall	May 4	Mar 27	Jun 11	Jun 3	Apr 23	Jun 30 [†]	31	16	3	73
Coastal	May 7 [a]	Mar 27	Jun 10	Jun 1 [e]	Apr 23	Jun 30 [†]	25 [h]	16	3	73
Brooks	Apr 28 [b]	Mar 31	May 24	Jun 6 [f]	May 16	Jun 30 [†]	39 [i]	12	15	64
W Coastal	May 6 [cd]	Mar 31	Jun 11	Jun 2 [g]	Apr 23	Jun 30 [†]	26 [jk]	14	3	66
E Coastal	May 10 [c]	Mar 27	May 25	May 31 [g]	May 16	Jun 16	21 [j]	19	4	73
W Brooks	May 1 [cd]	Apr 18	May 21	Jun 9 [g]	May 25	Jun 21	39 [kl]	8	26	47
E Brooks	Apr 28 [d]	Mar 31	May 24	Jun 6 [g]	May 16	Jun 30 [†]	39 [l]	13	15	64

Start date, end date, and duration of migration are reported at three scales: overall, by coarse wintering area, and by fine wintering area. Values with different superscript letters had statistically significant ($p < 0.05$) differences in timing. Comparisons were only done within each timing metric and scale, not between metrics or scales.

[†] June 30 was designated as the end of an analysis-year. Therefore, if a caribou's migratory movements were not ended by June 30, that date was assigned as their end date of migration.

S4 Table. Path distances (km) for Teshekpuk Caribou Herd fall and spring migration.

	Fall migration				Spring migration			
	$\bar{x}$	sd	min	max	$\bar{x}$	sd	min	max
Overall	423	204	42	1078	227	146	10	624
Coastal	359 [a]	182	42	1078	132 [f]	84	10	427
Brooks	525 [b]	197	125	1028	369 [g]	95	122	624
W Coastal	401 [c]	181	52	1078	150 [h]	86	25	427
E Coastal	244 [d]	125	42	469	84 [h]	57	10	207
W Brooks	656 [e]	69	548	717	451 [i]	78	336	624
E Brooks	492 [ce]	205	125	1028	349 [i]	88	122	500

Path distance was calculated for caribou-years classified as "migration" (n = 129) by summing the distances between consecutive locations during an individual's migration period (as determined by the first passage time – net squared displacement method) on datasets standardized to have at most two locations per day. Patterns were similar for Euclidean distance (S2 Appendix: S5 Table). Distances are reported at three scales: overall, by coarse wintering area, and by fine wintering area. Values with different superscript letters had statistically significant ($p < 0.05$) differences in migration distance. Comparisons were only done within each season and scale, not between scales.

S5 Table. Euclidean distances (km) for Teshekpuk Caribou Herd fall and spring migration.

	Fall migration					Spring migration				
	$\bar{x}$		sd	min	max	$\bar{x}$		sd	min	max
Overall	179		111	23	492	163		113	8	489
Coastal	113	a	64	23	275	91	e	61	8	300
Brooks	284	b	87	111	492	271	f	81	79	489
W Coastal	120	c	70	23	275	105	g	64	18	300
E Coastal	92	c	39	31	173	51	g	27	8	117
W Brooks	416	d	72	218	492	389	h	58	310	489
E Brooks	250	d	51	111	389	241	h	54	79	349

Values report the Euclidean distance between individual migration start and end locations (as determined by the first passage time – net squared displacement method), using datasets standardized to have at most two locations per day, for caribou-years classified as “migration” (n = 129). Distances are reported at three scales: overall, by coarse wintering area, and by fine wintering area. Values with different superscript letters had statistically significant ($p < 0.05$) differences in migration distance. Comparisons were only done within each season and scale, not between scales.

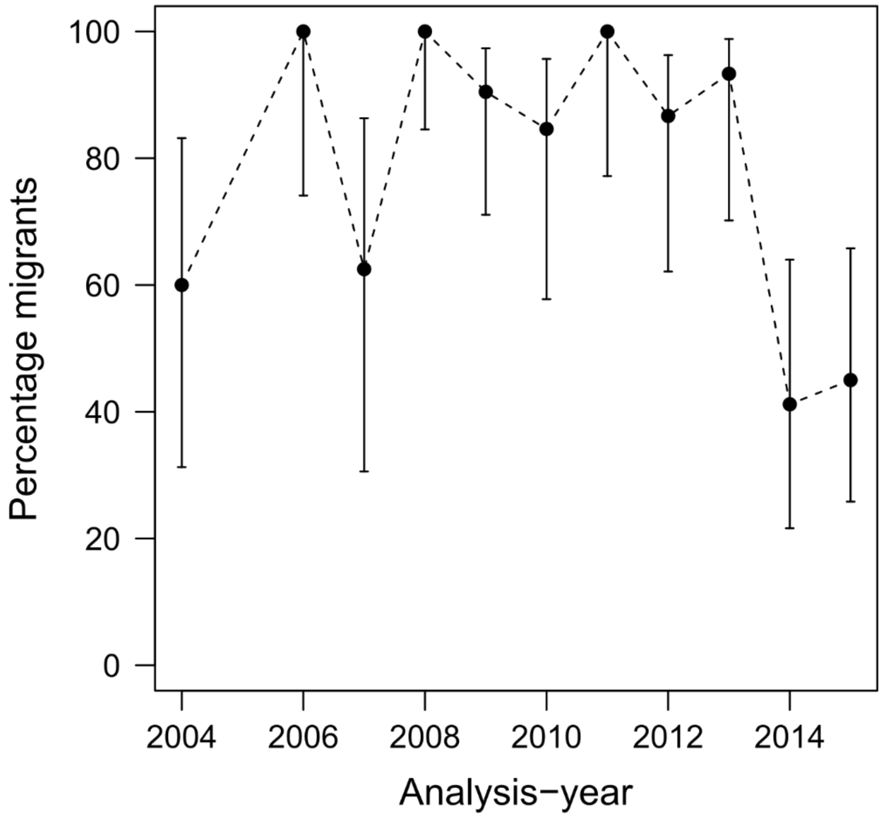

S1 Fig. Percentage of caribou classified as migrants in each analysis-year by the first passage time – net squared displacement (FPT-NSD) analysis. Analysis-years begin 1 July of the indicated year and continue through 30 June of the following year. Confidence intervals were calculated using the Wilson method from the binom R package [121].

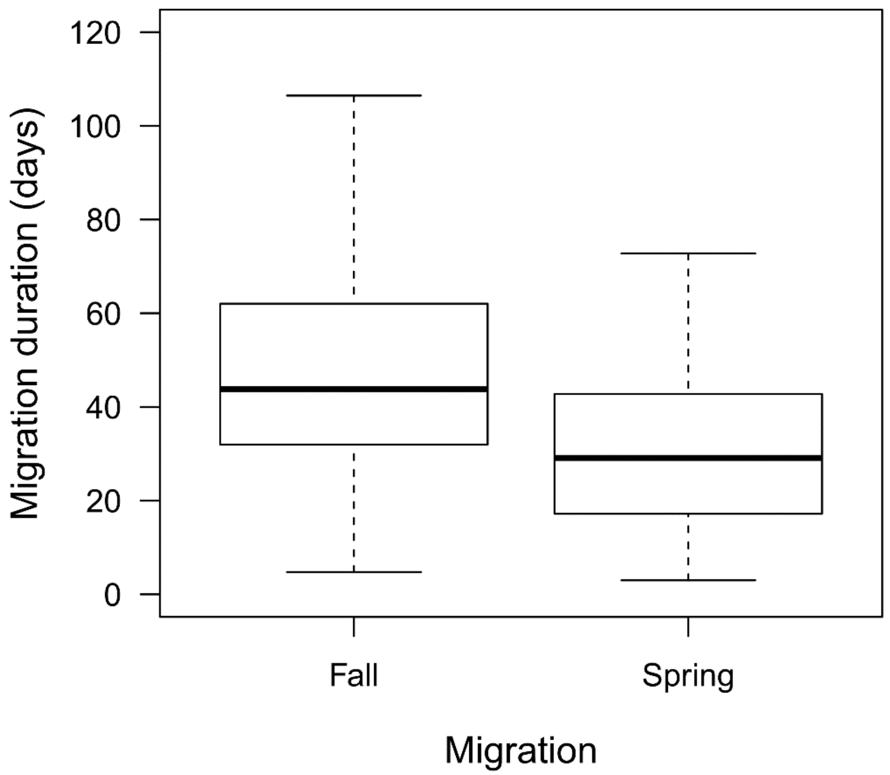

S2 Fig. Duration of fall and spring migration for the Teshekpuk Caribou Herd. Across all migrants ($n = 129$), fall migration tended to take longer than spring migration ($p < 0.001$).

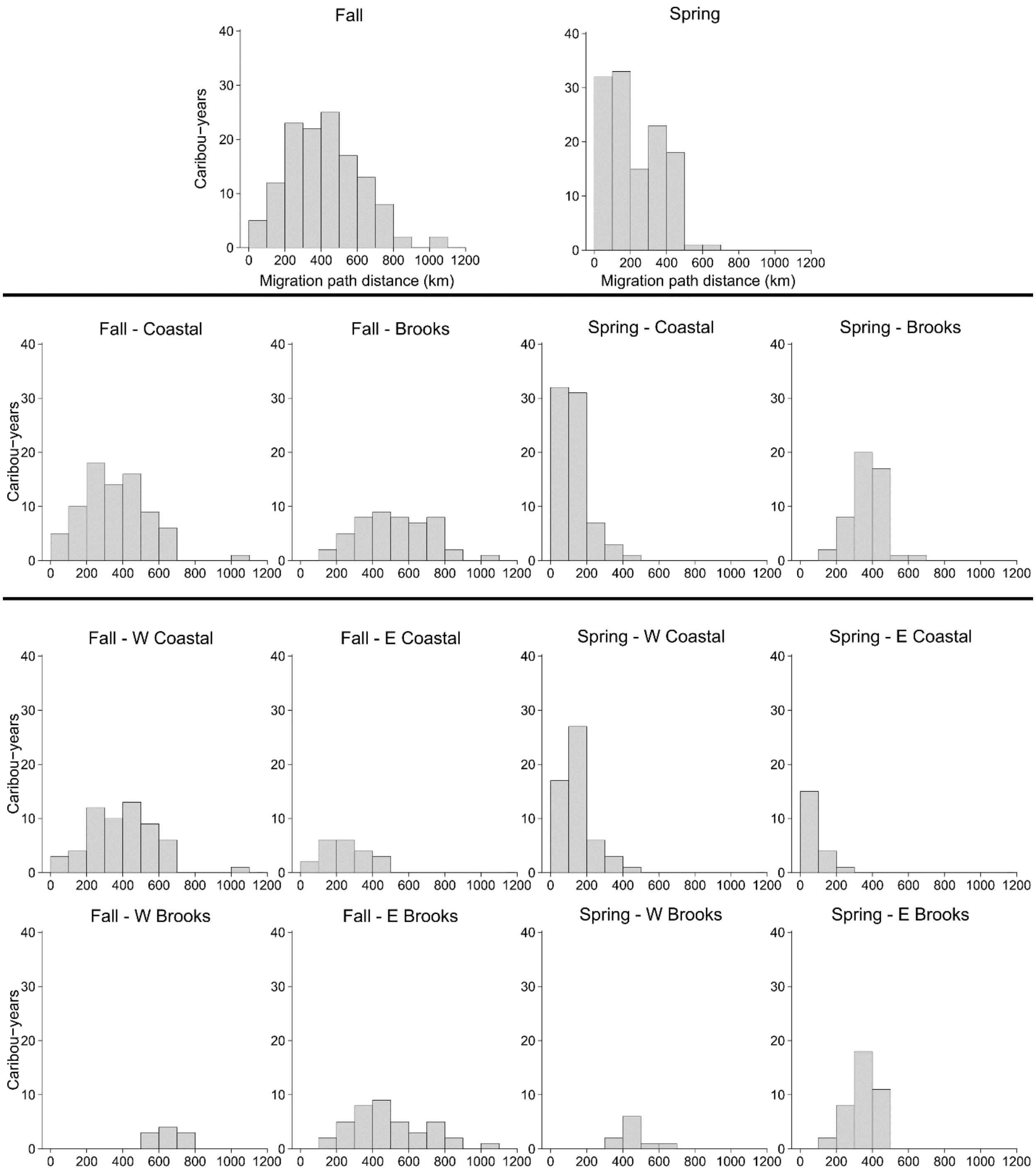

S3 Fig. Migration path distance (km) by season. Data shown combined for all caribou-years (top row), divided at a coarse scale – coastal plain versus Brooks Range (second row), and divided at a fine scale – four wintering areas (bottom two rows). Path distance was calculated for caribou-years classified as "migration" (n = 129) by summing the distances between consecutive locations during an individual's migration period (as determined by the first passage time – net squared displacement method) on datasets standardized to have at most two locations per day. See S2 Appendix: S4 Fig for results using Euclidean distance of migration.

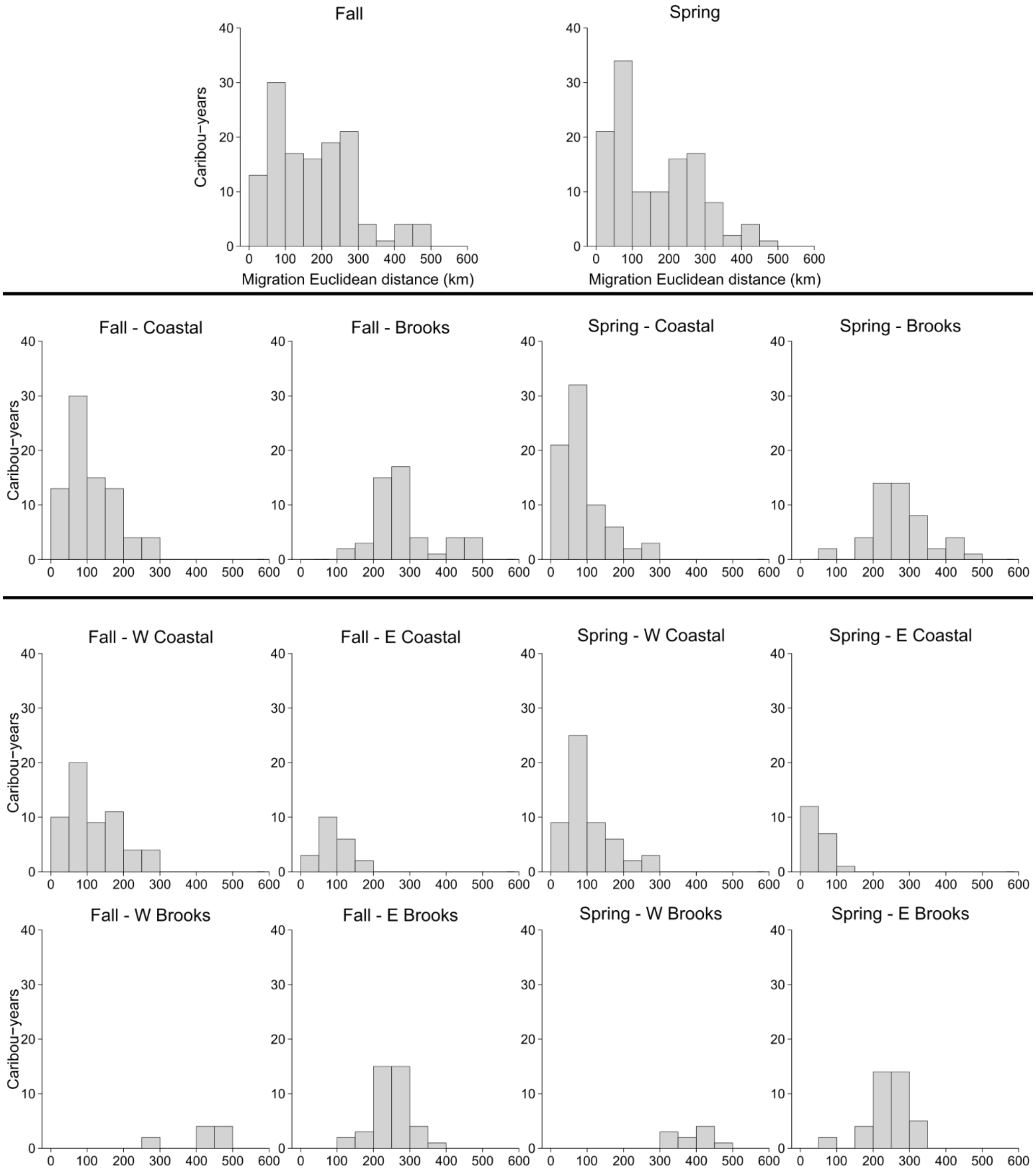

S4 Fig. Euclidean distance of migration (km) by season. Data shown combined for all caribou-years (top row), divided at a coarse scale – coastal plain versus Brooks Range (second row), and divided at a fine scale – four wintering areas (bottom two rows). Data are depicted for caribou-years classified as “migration” (n = 129) by the first passage time – net squared displacement method. See S2 Appendix: S3 Fig for path distance of migration.

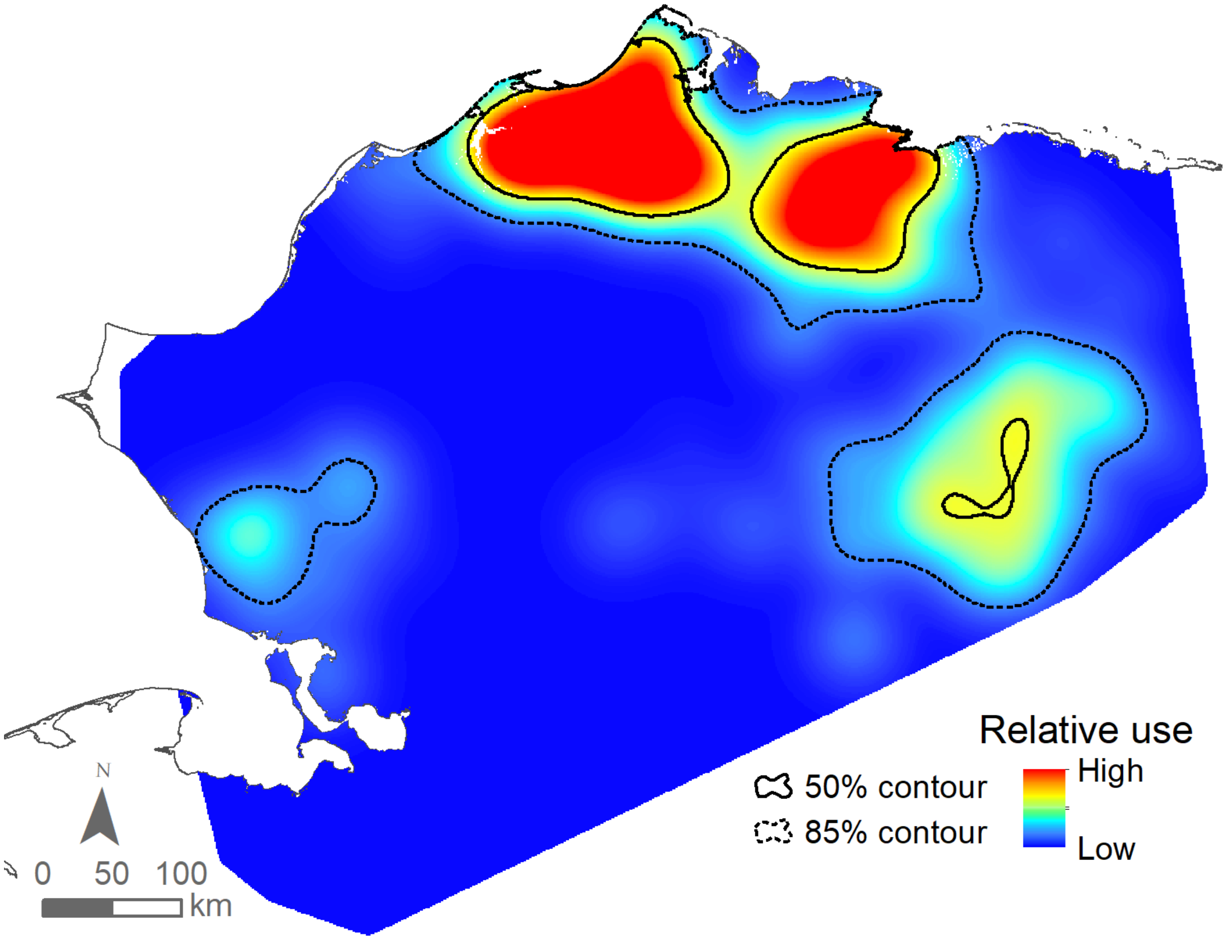

S5 Fig. Winter population-level utilization distribution for the Teshekpuk Caribou Herd. We used the 50% contours (solid black lines) to distinguish wintering areas in the high-density coastal plain region and 85% contours (dashed black lines) in the lower density Brooks Range region. See Fig 1 for a depiction of final wintering areas.

References

121. Dorai-Raj S. binom: Binomial confidence intervals for several parameterizations. R package version 1.1-1. 2014. Available: https://CRAN.R-project.org/package=binom
