# Supplementary material for: Variation in winter site fidelity within and among individuals influences movement behavior in a partially migratory ungulate
Source: PLoS One. 2021 Sep 30;16(9):e0258128. doi: 10.1371/journal.pone.0258128 (PMC8483381; doi:10.1371/journal.pone.0258128)
Supplement: S3 Appendix — S1 Metadata. Description of the data files contained in S3 Appendix. S1 Data. Caribou movement data summary file. Summary.csv file containing data for the 164 caribou-years of Teshekpuk Caribou Herd movement data analyzed in this study. S2 Data. TCH winter population-level utilization distribution raster. Raster file in GeoTiff format depicting the population-level winter utilization distribution for the Teshekpuk Caribou Herd, as shown in S2 Appendix: S5 Fig. (ZIP) [file pone.0258128.s003.zip › S1Metadata.pdf]

Fullman, T.J., Person, B.T., Prichard, A.K., Parrett, L.S. 2021. Variation in winter site fidelity within and among individuals influences movement behavior in a partially migratory ungulate. PLOS ONE.

S3 Appendix: Data

File list (files found within S3Appendix.zip)

S1Metadata.pdf (this file)	Description of the data files contained in S3 Appendix.
S1Data.csv	Caribou movement data summary file.
S2Data.tif	Winter population-level utilization distribution raster.

Data description

S1 Data

This .csv file contains summary data for the 164 caribou-years of Teshekpuk Caribou Herd movement data analyzed in this study. Data in the file consist of the following fields:

id	Caribou-year identifier, consisting of the animal identification code and analysis-year start (e.g., 0203_2008).
year	Analysis-year start. Analysis-years begin 1 July of the indicated year and continue through 30 June of the following year.
fix_interval	Duration between GPS position acquisitions, in hours.
nobs	Total number of recorded observations during the given analysis-year.
start_date	Date and time of first recorded location for the given analysis-year in “month/day/year time” format. Time reported in in Alaska standard time.
end_date	Date and time of last recorded location for the given analysis-year in “month/day/year time” format. Time reported in in Alaska standard time.

mvmt_class	Movement class (migrant, resident, other) as assigned by the first passage time – net squared displacement (FPT-NSD) method. See main text for details.
winter_coarse	Coarse-scale indicator of winter use, reporting overwintering in either the coastal plain or Brooks Range.
winter_fine	Fine-scale indicator of winter use, reporting overwintering in one of the four wintering areas depicted in Fig 1. Individuals were assigned to the wintering area with which their individual-level utilization distribution had the greatest overlap. See main text for details.
fall_start, spring_start	Migration start date and time in "month/day/year time" format. Time reported in in Alaska standard time. Non-migrants were assigned a value of "NA."
fall_end, spring_end	Migration end date and time in "month/day/year time" format. Time reported in in Alaska standard time. Non-migrants were assigned a value of "NA."
fall_duration, spring_duration	Migration duration, in days. Non-migrants were assigned a value of "NA."
fall_dist_euc_km, spring_dist_euc_km	Euclidean distance of migration, in km. This was calculated using a standardized dataset where each caribou-year contributed at most two locations per day. Non-migrants were assigned a value of "NA."
fall_dist_path_km, spring_dist_euc_km	Path distance of migration, in km. Path distance was calculated by summing the distances between consecutive locations during an individual's migration period (as determined by the FPT-NSD method) on datasets standardized to have at most two locations per day. Non-migrants were assigned a value of "NA."
fall_directedness, spring_directedness	Directedness of migration metric. Directedness is calculated taking as the Euclidean distance of migration divided by the path distance. This yields a unitless value ranging between 0-1. It indicates the degree of tortuosity of a caribou's migration path, with lower values indicating a more tortuous migration path and higher values indicating more directed movement. A directedness value of 1 would indicate that the animal migrated in a straight line following the path of least distance during migration. Non-migrants were assigned a value of "NA."

S2 Data

Raster file in GeoTiff format depicting the population-level winter utilization distribution for the Teshekpuk Caribou Herd (S2 Appendix: S5 Fig). We combined all standardized 12-hour locations for each season and calculated a single seasonal population-level utilization distribution using kernel density estimation in the R package adehabitatHR [49], using the *ad hoc* approach of Kie [58] to select the optimal bandwidth.

Licensing

Data are copyright of the authors. Reuse is permitted following the same terms as the manuscript, following the Creative Commons Attribution License. This permits use, distribution, and reproduction, provided the original authors and associated manuscript are credited.

References

49. Calenge C. The package "adehabitat" for the R software: A tool for the analysis of space and habitat use by animals. Ecol Modell. 2006;197: 516–519. doi:10.1016/j.ecolmodel.2006.03.017

58. Kie JG. A rule-based ad hoc method for selecting a bandwidth in kernel home-range analyses. Anim Biotelemetry. 2013;1: 13. doi:10.1186/2050-3385-1-13
